# The role of inflammation and cardiovascular disease risk on microvascular and macrovascular endothelial function in patients with rheumatoid arthritis: a cross-sectional and longitudinal study

**DOI:** 10.1186/ar3847

**Published:** 2012-05-17

**Authors:** Aamer Sandoo, George D Kitas, Douglas Carroll, Jet JCS Veldhuijzen van Zanten

**Affiliations:** 1 Department of Rheumatology, Dudley Group NHS Foundation Trust, Pensnett Road, Dudley, DY1 2HQ, UK; 2 School of Sport and Exercise Sciences, University of Birmingham, Edgbaston, Birmingham, B15 2TT, UK; 3 Arthritis Research UK Epidemiology Unit, University of Manchester, Oxford Road, Manchester, M13 9PT, UK

## Abstract

**Introduction:**

Rheumatoid arthritis (RA) is associated with an increased risk for cardiovascular disease (CVD), and it has been postulated that RA disease-related inflammation contributes to endothelial dysfunction. The aim of the present work was to examine predictors (RA-related and CVD risk factors) and anti-tumor necrosis factor-alpha (anti-TNF-α) treatment effects on endothelial function in different vascular beds.

**Methods:**

Microvascular endothelial function (laser Doppler imaging with iontophoresis of acetylcholine and sodium-nitroprusside), and macrovascular endothelial function (flow-mediated dilatation and glyceryl-trinitrate-mediated dilatation) were analyzed in parallel with disease activity. Individual CVD risk factors and global CVD risk were assessed cross-sectionally in 99 unselected RA patients and longitudinally (baseline, 2 weeks, and 3 months) in 23 RA patients commencing anti-TNF-α therapy.

**Results:**

In this cross-sectional study, regression analyses revealed that markers of RA disease-related inflammation were not associated with microvascular or macrovascular endothelium-dependent function (*P *> 0.05); global CVD risk inversely correlated with microvascular endothelium-dependent function (*P *< 0.01) and with macrovascular endothelium-independent function (*P *< 0.01). In the longitudinal study, only microvascular endothelium-dependent function showed an improvement after 2 weeks of anti-TNF-α treatment when compared with baseline (437% ± 247% versus 319% ± 217%; *P *= 0.001), but no association was evident between change in endothelial function and change in inflammatory markers.

**Conclusions:**

Classical CVD risk may influence endothelial function more than disease-related markers of inflammation in RA. Classical CVD risk factors and anti-TNF-α medication have different effects on microvascular and macrovascular endothelial function, suggesting that combined CVD-prevention approaches may be necessary. Prospective studies examining whether assessments of vascular function are predictive of long-term CV outcomes in RA are required.

## Introduction

Rheumatoid arthritis (RA) is a chronic inflammatory musculoskeletal disease that affects ~0.8% of the adult population. RA also associates with an increased risk for cardiovascular disease (CVD) [1], which is only partially explained by traditional CVD risk factors [2]. The inflammatory processes of RA and CVD are remarkably similar, suggesting that RA disease-related inflammation might contribute to the excess CVD risk [3].

The vascular endothelium is responsible for maintaining an atheroprotective environment through the release of vasoactive factors, particularly nitric oxide (NO). Noninvasive assessments of peripheral endothelial function in the microcirculation and the macrocirculation may predict adverse cardiovascular outcomes in patients at risk of developing, or with prevalent CVD [4]. Endothelial cells differ in structure and phenotype depending on vessel type [5], and heterogeneous responses to *in vitro *stimulation are displayed in different vascular beds [6], suggesting that endothelial dysfunction (ED) may occur differentially in different vascular beds [6]. Evidence suggests that coronary microvascular disease is apparent in the absence of macrovascular disease in RA [7], and we previously showed that microvascular and macrovascular endothelial function are independent of each other in this population [8]. These findings highlight the importance of assessing endothelial function in different vascular beds.

A number of studies have reported the presence of microvascular and macrovascular ED in RA patients relative to age- and sex-matched healthy controls, which can be improved after treatment with antiinflammatory medications (such as anti-tumor necrosis factor-alpha (anti-TNF-α) [9]. Even though an association between endothelial function and inflammation is often assumed, surprisingly few studies have actually examined this [9]. Cross-sectional studies report equivocal findings; some find an association between inflammatory markers and endothelial function [10], whereas others do not [11]. Longitudinal studies revealed no correlation between changes in inflammatory markers and changes in endothelial function [12,13], and the improvement in microvascular endothelial function after treatment occurred without any change in inflammatory markers [14]. Thus, it remains possible that endothelial function in RA is determined by factors other than systemic inflammation, such as CVD risk factors [9].

The prevalence of classical CVD risk factors is increased in patients with RA [2], and their control is worse [15], compared with that of the general population [16]. At present, no studies have examined associations between classical CVD risk factors in the microcirculation, and only a very few studies have been conducted in the macrocirculation, with inconsistent findings [9]. CVD risk can be calculated by incorporating individual CVD risk factors into algorithms to yield "global" CVD risk scores. Examples of such algorithms are the Framingham Risk Score (FRS) [17] and the Systematic Coronary Risk Evaluation (SCORE) [18]. However, these algorithms do not account for systemic inflammation, whereas the more recent Reynolds risk score does, as it includes C-reactive protein (CRP) in the algorithm. In addition, QRISK2 is the only CVD-risk algorithm that incorporates the presence of RA, as well as socioeconomic status and ethnicity [19]. To our knowledge, the relation between these risk algorithms and microvascular and macrovascular endothelial function has not been investigated in RA.

The objectives of the present studies were to examine determinants of microvascular and macrovascular endothelial function in RA, with a specific focus on disease-related markers of inflammation and classical CVD risk.

## Materials and methods

### Cross-sectional study

Ninety-nine consecutive rheumatoid arthritis (RA) patients were recruited from the rheumatology outpatient clinics of the Dudley Group of Hospitals NHS Trust, United Kingdom. All patients met the retrospective application of the 1987 revised RA criteria of the American College of Rheumatology [20]. Patients were excluded if they had previously confirmed acute coronary syndrome or established cardiovascular disease (CVD).

### Longitudinal study

Twenty-three RA patients from study one, who were about to start anti-TNF-α therapy on clinical indication (UK guidelines), were recruited into a 3-month prospective study. The patients had not previously received any other biologic drugs, including anti-TNF-α. Patients were assessed before starting anti-TNF-α (pre-treatment), and 2 weeks and 3 months after initiation of treatment. Fifteen (65%) of the anti-TNF-α patients were started on 40 mg of adalimumab, six (26%) on 50 mg of etanercept, and two (9%) on infliximab with a dosage of 3 mg/kg. Patients continued treatment with other drugs during the study period, and these included 16 (70%) patients taking methotrexate, one (4%) taking cyclooxygenase II inhibitors, six (26%) taking nonsteroidal antiinflammatory drugs, six (26%) taking prednisolone, four (18%) taking anti-hypercholesterolemic drugs, and five (22) taking antihypertensive drugs. No change in any of these medications or their doses occurred during the follow-up period. Assessments at 2 weeks and 3 months were performed before receiving the next dose of the anti-TNF-α drug.

The study had ethics committee approval, and all participants provided written informed consent.

### Study protocol

Patients reported to a thermoregulated (22°C ± 0.9°C) vascular laboratory after a 12-hour overnight fast. They were asked to refrain from exercise 24 hours before the session, and from smoking, 12 hours before the session. For ethical reasons, drug regimens were not interrupted. All participants underwent a detailed clinical examination, and demographic information was collected with a questionnaire. The disease activity score (DAS28) [21] and the Stanford Health Assessment Questionnaire (HAQ) [22] were completed, along with the patients global CVD risk scores. Blood pressure measurements were taken by using an automated blood pressure monitor (Datascope Accutor, Mahwah, NJ, USA). A blood sample was also obtained. Patients in the cross-sectional study were assessed at one time point only, whereas patients in the longitudinal study were assessed at pre-treatment baseline, and at 2 weeks and 3 months after treatment with anti-TNF-α.

### Blood sampling

The serum was analyzed for total cholesterol, high-density lipoprotein cholesterol (HDL), triglycerides, CRP, and glucose by using a Vitros 5.1 FS Chemistry system. Westergren erythrocyte sedimentation rate (ESR) was measured by using the Starrsed Compact (Mechatronics BV, Hoorn, The Netherlands). Insulin was estimated from serum stored at -20°C by using an Immulite 2500 analyzer (Diagnostic Products Corporation, California, USA). Insulin sensitivity was assessed by calculating the Homeostasis Model Assessment Insulin Resistance (HOMA IR) and Quantitative Insulin Sensitivity Check Index (QUICKI), as previously described [23,24]. All tests were carried out in the routine and research laboratories of Dudley Group NHS Foundation Trust.

### Global CVD risk

Four separate global CVD-risk algorithms were used: FRS [25], SCORE [18], Reynolds risk score [26], and the QRISK2 [19]. These algorithms were calculated at baseline for patients in the cross-sectional and longitudinal studies.

### Microvascular endothelial function

Endothelial function of the microvasculature was assessed non-invasively by using LDI (Moor LDI 2 SIM; Moor Instruments Ltd, Devon, UK) with iontophoresis of 1% acetylcholine (ACh, endothelium-dependent) and 1% sodium-nitroprusside (SNP, endothelium-independent) (Sigma Chemical Co, Montvale, NJ, USA) in 2.5 ml solution containing 0.5% saline, by a single observer (AS). The technique was performed according to previously established guidelines [27] and has been described in detail previously [28]. This technique has an intraobserver coefficient of variation (CV) for ACh and SNP of 6.5% and 5.9%, respectively, for AS.

### Macrovascular endothelial function

Assessment of macrovascular endothelium-dependent function was performed by using flow-mediated dilatation (FMD) with high-resolution ultrasonography of the brachial artery (Acuson Antares ultrasound system; Siemens PLC, Camberley, UK) according to previously established guidelines [29]. Endothelium-independent responses were examined by administration of a 500-μg sublingual glyceryl-trinitrate (GTN) tablet (Alpharma, Barnstaple, UK). The intraobserver CV for the study ultrasonographer (AS) was 10.7% for FMD and 11.8% for GTN assessments, respectively. For all vascular tests, endothelial function was expressed as the percentage increase in perfusion or diameter from baseline, and all analysis was carried out offline by AS, who was blinded to the identity of the patient.

### Statistical analysis

Statistical analysis was performed by using SPSS15 (SPSS Inc., Chicago, IL, USA). Variables were tested for normality by the Kolmogorov-Smirnov test. Means and standard deviations (SD) were calculated for normally distributed continuous variables, and proportions for categorical variables. Non-normally distributed data were presented as median (25^th ^to 75^th ^percentile values). Log transformation was performed for positively skewed variables, as appropriate. Variations in degrees of freedom reflect occasionally missing data.

#### Cross-sectional study

To assess determinants of vascular function, univariate linear regression (continuous variables) and logistic regression (dichotomous variables) was used. Inflammatory markers, global CVD risk, and CVD risk factors were entered as independent variables, with each measure of vascular function entered separately as the dependent variable. Step-wise multiple regression analysis was conducted to determine independent predictors of endothelial function, and only those variables that came out as significantly associated with endothelial function in the univariate analyses were entered into this analysis. The entry probability was 0.05, and none of the variables were forced back into the model.

#### Longitudinal study

Changes (Δ) in each parameter of endothelial function, CVD risk factors, and disease-related markers of systemic inflammation were assessed by using three Time (pre-treatment baseline, 2 weeks, and 12 weeks) repeated-measures analysis of variance (ANOVA). Where appropriate, Fisher LSD *post hoc *tests were used for pair-wise comparisons. Endothelial function did not differ between the three different types of anti-TNF-α treatment at any time point (data not shown); therefore, all treatments were analyzed together. The Δ in endothelial function and disease-related parameters was calculated by subtracting the pretreatment baseline values from the values obtained at 2 and 12 weeks, respectively. Linear regression was used to examine whether changes in endothelial function were related to changes in disease-related inflammatory markers at each time point.

## Results

### Cross-sectional study

#### Patient characteristics

The general and disease-related characteristics for patients in the cross-sectional study are presented in Table 1. Individual CVD risk factors as well as the global CVD risk scores for these patients are shown in Table 2.

**Table 1:** General and disease characteristics as well as endothelial function scores for patients in the cross-sectional study

	RA patients	Number
General characteristics		
Sex female *n *(%)	72 (73)	99
Disease characteristics		
RF positive *n *(%)	70 (78)	90
Disease duration (years)	11 ± 10	74
ESR (mm/hr)	17.0 (8.8-28.3)	90
CRP (mg/L)	5.0 (2.9-13.50)	93
DAS28	3.6 ± 1.3	93
HAQ	1.7 ± .87	95
RA medications		
Methotrexate *n *(%)	60 (60)	99
Prednisolone *n *(%)	22 (22)	99
NSAIDS *n *(%)	18 (18)	99
Cyclooxygenase II inhibitors *n *(%)	11 (11)	99
Anti-TNF-α therapy *n *(%)	11 (11)	99
CVD medications		
Antihypertensive *n *(%)	25 (25)	99
Antihypercholesterolemic *n *(%)	12 (12)	99
Beta-blocker *n *(%)	7 (7)	99
Calcium channel blocker *n *(%)	5 (5)	99
Microvascular function		
Endothelium-dependent (ACh%)	236 (152-407)	94
Endothelium-independent (SNP%)	261 (181-384)	94
Macrovascular function		
Endothelium-dependent (FMD%)	9.5 (4.8-13.5)	96
Endothelium-independent (GTN%)	24.0 (16.3-30.4)	93

Results are expressed as number (percentage), mean ± SD, or median (25^th ^to 75^th^) percentile, as appropriate. Ach, acetylcholine; CRP, C-reactive protein; CVD, cardiovascular disease; DAS28, disease activity score in 28 joints; ESR, erythrocyte sedimentation rate; FMD, flow-mediated dilatation; GTN, glyceryl trinitrate; HAQ, Health Assessment Questionnaire, NSAID, nonsteroidal antiinflammatory drug; RA, rheumatoid arthritis; RF, rheumatoid factor; TNF, tumor necrosis factor; SNP, sodium nitroprusside.

**Table 2:** Individual CVD risk factors and global CVD risk scores for patients in the cross-sectional study

	RA patients	Number
Individual CVD risk factors		
Age (years)	56 ± 12	99
BMI (kg/m^2^)	30 ± 6	99
SBP (mm Hg)	133 ± 16	96
DBP (mm Hg)	81 ± 10	96
Total cholesterol (m*M*)	5.1 ± 1.0	93
HDL-C (m*M*)	1.5 ± 0.3	93
Triglycerides (m)	1.5 ± 0.7	93
TC/HDL ratio	3.5 ± 8.5	93
Glucose (m*M*)	4.6 (4.3-4.9)	91
Insulin (p*M*)	70.4 (40.6-105.5)	89
HOMA IR	2.1 (1.1-3.2)	87
QUICKI	0.35 ± 0.41	87
Cigarette-smoking status		
Never smoked	38 (42)	91
Previous smokers	36 (40)	91
Current smokers	17 (19)	91
Global CVD risk scores		
Framingham Risk Score	5 (3-10)	87
TC SCORE	1 (0-2)	64
TC/HDL SCORE	1 (0-2)	64
Reynolds Risk Score	8 (3-14)	65
QRISK 2	15 (7-26)	95

Results are expressed as median (25^th ^to 75^th ^percentile values) or mean ± SD, as appropriate. BMI, body mass index; DBP, diastolic blood pressure; HDL-C, high-density lipoprotein cholesterol; HOMA IR, homeostasis model assessment insulin resistance; QUICKI, quantitative insulin sensitivity check index; SBP, systolic blood pressure; SCORE, systematic coronary risk evaluation; TC, total cholesterol.

#### Microvascular function

##### Endothelium-dependent and endothelium-independent function

###### Disease-related markers of inflammation

No association was apparent between microvascular endothelium-dependent or endothelium-independent function and any of the inflammatory parameters (ESR, CRP, DAS28, or disease duration) (Table 3).

**Table 3:** Linear regression analysis for general and RA disease-related characteristics and endothelial function in RA patients from the cross-sectional study

	Microvascular function		Macrovascular function	
	**Endothelium-dependent**	**Endothelium- independent**	**Endothelium- dependent**	**Endothelium- independent**

RA disease-related characteristics				
Disease duration	*β *= -0.12, *P *= 0.29	*β *= 0.21, *P *= 0.07	*β *= -0.04 *P *= 0.76	*β *= -0.12, *P *= 0.34
LogCRP	*β *= -0.61, *P *= 0.05	*β *= 0.10, *P *= 0.32	*β *= 0.15, *P *= 0.13	*β *= 0.00, *P *= 0.97
LogESR	*β *= -0.09, *P *= 0.41	*β *= 0.18, *P *= 0.10	*β *= -0.01, *P *= 0.95	*β *= -0.08, *P *= 0.46
DAS28	*β *= 0.15, *P *= 0.15	*β *= 0.02, *P *= 0.84	*β *= 0.06, *P *= 0.59	*β *= -0.01, *P *= 0.93
General characteristics				
Age	*β *= -0.37, *R*^2 ^= 0.135^c^	*β *= -0.20, *R^2 ^= 0*.042^a^	*β *= -0.24, *R*^2 ^= 0.55^a^	*β *= -0.36, *R*^2 ^= 0.126^c^
BMI	*β *= 0.21, *P *= 0.05	*β *= 0.08, *P *= 0.41	*β *= -0.12, *P *= 0.26	*β *= -0.21, *P *= 0.05
Resting SBP	*β *= -0.11, *P *= 0.30	*β *= -0.02, *P *= 0.88	*β *= -0.11, *P *= 0.27	*β *= -0.27, *R*^2 ^= 0.066^b^
Resting DBP	*β *= 0.04, *P *= 0.67	*β *= 0.08, *P *= 0.44	*β *= -0.12, *P *= 0.25	*β *= -0.05, *P *= 0.61

RA disease-related characteristics and general characteristics were entered as independent variables, whereas microvascular and macrovascular endothelial functions were entered as dependent variables in the regression analysis. BMI, body mass index; CRP, C-reactive protein; DAS28, disease activity score in 28 joints; DBP, diastolic blood pressure; ESR, erythrocyte sedimentation rate; SBP, systolic blood pressure. ^a^*P *< 0.05; ^b^*P *< 0.01; ^c^*P *< 0.001. *R^2 ^*value is shown for all significant associations only.

###### Individual classical CVD risk factors

Both microvascular endothelium-dependent and endothelium-independent function were inversely correlated with age only (see Tables 3 and 4).

**Table 4:** Linear and binary regression between classical CVD risk and endothelial function in RA patients from the cross-sectional study

	Microvascular function		Macrovascular function	
	**Endothelium-dependent**	**Endothelium- independent**	**Endothelium- dependent**	**Endothelium- independent**

CVD risk factors				
Insulin	*β *= 0.13, *P *= 0.23	*β *= 0.04, *P *= 0.73	*β *= -0.04, *P *= 0.71	*β *= -0.25, *R*^2 ^= 0.065^a^
HOMA	*β *= 0.09, *P *= 0.41	*β *= -0.01, *P *= 0.95	*β *= -0.06, *P *= 0.61	*β *= -0.23, *R*^2 ^= 0.052^a^
QUICKI	*β *= -0.07, *P *= 0.53	*β *= -0.02, *P *= 0.88	*β *= 0.11, *P *= 0.30	*β *= 0.30, *R*^2 ^= 0.095^b^
High cholesterol	OR = 1.01 (0.99-1.00)	OR = 1.00 (0.99-1.00)	OR = 1.08 (0.99-1.17)	OR = 1.09 (1.02-1.17)^a^
Hypertension	OR = 1.00 (1.00-1.00)	OR = 1.00 (0.99-1.00)	OR = 1.08 (0.99-1.17)	OR = 1.07 (1.01-1.14)^a^
Cigarette smoking	OR = 1.00 (1.00-1.00)	OR = 1.00 (1.00-1.00)	OR = 1.04 (0.97-1.13)	OR = 1.04 (0.99-1.10)
Global CVD risk				
FRS	*β *= -0.26, *R*^2 ^= 0.068^b^	*β *= -0.18, *P *= 0.09	*β *= -0.12, *P *= 0.26	*β *= -0.32, *R*^2 ^= 0.101^b^
TC score	*β *= -0.28, *R*^2 ^= 0.077^a^	*β *= -0.17, *P *= 0.18	*β *= -0.23, *P *= 0.08	*β *= -0.14, *P *= 0.30
TC/HDL score	*β *= -0.26, *R*^2 ^= 0.068^a^	*β *= -0.17, *P *= 0.19	*β *= -0.25, *P *= 0.05	*β *= -0.17, *P *= 0.18
Reynolds Risk Score	*β *= -0.30, *R*^2 ^= 0.088^a^	*β *= -0.12, *P *= 0.37	*β *= -0.27, *R*^2 ^= 0.072^a^	*β *= -0.51, *R*^2 ^= 0.259^b^
QRISK 2	*β *= -0.33, *R*^2 ^= 0.109^b^	*β *= -0.19, *P *= 0.08	*β *= -0.22, *R*^2 ^= 0.048^a^	*β *= -0.51, *R*^2 ^= 0.255^b^

CVD risk factors and global CVD risk scores were entered as independent variables, and endothelial functions, as dependent variables in the linear and binary regression analysis. Odds ratio (OR) with 95% confidence interval is presented for all binary regression analysis. CVD, cardiovascular disease; FRS, Framingham risk score; HDL, high-density lipoprotein; HOMA, homeostasis model assessment; QUICKI, quantitative insulin sensitivity check index; SCORE, systematic coronary risk evaluation; TC, total cholesterol. ^a^*P *< 0.05; ^b^*P *< 0.01. R^2 ^value is shown for all significant associations only.

###### Global CVD risk

The FRS, SCORE, Reynolds risk score, and QRISK2 were all inversely associated with microvascular endothelium-dependent function (Table 4), but no associations were evident for microvascular endothelium-independent function.

#### Macrovascular function

##### Endothelium-dependent and endothelium-independent function

###### Disease-related markers of inflammation

None of the inflammatory parameters (ESR, CRP, DAS28, or disease duration) was associated with macrovascular endothelium-dependent or endothelium-independent function (Table 3).

###### Individual classical CVD risk factors

Age was the only individual CVD risk factor associated with both macrovascular endothelium-dependent and endothelium-independent function (see Tables 3 and 4). In addition, resting SBP, insulin, HOMA, and QUICKI were associated with macrovascular endothelium-independent function. A modest association was noted between hypercholesterolemia, hypertension, and macrovascular endothelium-independent function (Tables 3 and 4). Step-wise multiple regression analysis revealed that QUICKI (*β *= 0.28; *R*^2 ^= 0.052; *P *< 0.01) and SBP (*β *= -0.13; *R*^2 ^= 0.052; *P *< 0.05) were independently associated with macrovascular endothelium-independent function.

###### Global CVD risk

Macrovascular endothelium-dependent function was inversely associated with Reynolds risk score and QRISK2, whereas macrovascular endothelium-independent function was associated with FRS, Reynolds risk score, and QRISK2 (Table 4).

### Longitudinal study

#### Patient characteristics

Patients in the longitudinal study, had an age (mean ± standard deviation) of 54 ± 15 years; 15 (65%) were female patients; 20 (87%) were rheumatoid factor positive; and the disease duration was 11 ± 11 years.

Eleven (48.8%) patients were never smokers; seven (30%), previous smokers; and five (22%) were current smokers.

#### Longitudinal effect of anti-TNF-α treatment on disease-related inflammation

The repeated measures ANOVA revealed an overall effect for morning stiffness, CRP, ESR, DAS28, and HAQ (Table 5). *Post hoc *analyses revealed that morning stiffness, CRP, DAS28, and HAQ were reduced after 2 weeks and remained low after 12 weeks of treatment. ESR improved 2 weeks after commencing treatment, but was similar to baseline levels at 12 weeks. None of the parameters differed significantly between weeks 2 and 12.

**Table 5:** Disease-related characteristics at baseline, 2 weeks, and 12 weeks

	Baseline	2 weeks	12 weeks	Treatment effect *F *(2, 22) =
Morning stiffness (min)	116 ± 75	72 ± 81^a^	55 ± 83^a^	4.33, *P *= 0.02
CRP (mg/L)	10 (4-14)	3 (2.9-6)^a^	5 (2.9-10)^a^	12.89, *P *< 0.001
ESR (mm/hr)	16 (9-34)	10 (5-21)^a ^	17 (5-27)	4.98, *P *= 0.01
DAS28	4.17 ± 0.96	2.74 ± 1.4^a ^	2.64 ± 1.07^a^	15.92, *P *< 0.001
HAQ	2.1 ± 0.5	1.3 ± 0.9^a ^	1.3 ± 0.9^a^	17.18, *P *< 0.001

Results are expressed as median (25^th ^to 75^th ^percentile values) or mean ± standard deviation, as appropriate. ^a^Different from baseline. CRP, C-reactive protein; DAS28, disease activity score; ESR, erythrocyte sedimentation rate; HAQ, Health Assessment Questionnaire.

#### Longitudinal effect of anti-TNF-α treatment on classical CVD risk

ANOVAs revealed an overall Time effect for HDL cholesterol (*F *(2, 22) = 4.98; *P < 0*.01), SBP (*F *(2, 22) = 5.63, *P < 0*.01) and DBP (*F *(2, 22) = 6.99; *P *< 0.01). *Post hoc *analysis showed that HDL cholesterol was higher at 2 weeks, but returned to baseline levels by 12 weeks. No change was found in the TC/HDL ratio with anti-TNF-α treatment. SBP and DBP were lower at 2 and 12 weeks relative to baseline. No change was noted in any other parameters.

#### Longitudinal effect of anti-TNF-α treatment on microvascular function

ANOVA revealed an overall Time effect for microvascular endothelium-dependent function after treatment (Table 6). *Post hoc *analysis confirmed that microvascular endothelium-dependent function was increased at 2 weeks, but not different from baseline at 12 weeks. No change in microvascular endothelium-independent function was found after treatment.

**Table 6:** Endothelial function during treatment in the longitudinal study

	Baseline	2 weeks	12 weeks	Treatment effect	Degrees of freedom
Microvascular function					
Endothelium-dependent (Ach%)	314 ± 214	423 ± 250^a^	348 ± 209^b^	*F *= 5.09, *P *= 0.01	2, 21
Endothelium-independent (SNP%)	247 ± 126	284 ± 147	261 ± 152	*F *= 1.18, *P *= 0.32	2, 21
Macrovascular function					
Endothelium-dependent (FMD%)	9.4 ± 6.8	12.0 ± 10.0	12.0 ± 8.1	*F *= 1.60, *P *= 0.21	2, 19
Endothelium-independent (GTN%)	22 ± 7.4	23 ± 7.2	24 ± 7.2	*F *= 0.31, *P *= 0.74	2, 19

Results are expressed as mean ± standard deviation. ^a^Different from baseline. ^b^Different from 2 weeks. Ach, acetylcholine; FMD, flow-mediated dilatation; GTN, glyceryl trinitrate-mediated dilatation; SNP, sodium nitroprusside.

#### Longitudinal effect of anti-TNF-α treatment on macrovascular function

Both macrovascular endothelium-dependent and endothelium-independent function did not change in response to anti-TNF-α treatment.

#### Association between changes in inflammation, CVD risk factors, and endothelial function

##### Microvascular function

###### Endothelium-dependent and endothelium-independent function

Linear regression analysis revealed that changes in inflammatory parameters and CVD risk factors were not correlated with change in microvascular endothelium-dependent or endothelium-independent function at 2 weeks or at 12 weeks (*P *> 0.10).

##### Macrovascular function

###### Endothelium-dependent and endothelium-independent function

In the macrovasculature, ΔESR and ΔDAS28 were not related to change in macrovascular endothelium-dependent function at 2 and 12 weeks. However, ΔCRP was associated with change in macrovascular endothelium-dependent function at 2 weeks (*β *= 0.52; *R*^2 ^= 0.269; *P *< 0.01), but not at 12 weeks. No associations were seen between change in CVD risk factors and macrovascular endothelium-dependent and -independent function at any time point.

##### Baseline inflammation and changes in endothelial function

Baseline ESR associated with the change in microvascular endothelium-dependent function at 2 weeks (*β *= 0.48; *R*^2 ^= 0.198; *P *< 0.05) and baseline CRP was associated with change in microvascular endothelium-dependent function at 2 weeks (*β *= 0.47; *R*^2 ^= 0.185; *P *< 0.05) and at 12 weeks (*β = 0*.42; *R*^2 ^= 0.138; *P *< 0.05). This suggests that those with high levels of inflammation at baseline showed the greatest improvement in microvascular endothelium-dependent function. No other significant correlations emerged from these analyses. The same analyses were repeated for the global CVD risk algorithms, but no associations were apparent.

## Discussion

These findings revealed that, in the cross-sectional study, disease-related markers of systemic inflammation were not associated with microvascular or macrovascular endothelial function in RA. Similarly, changes in inflammatory markers did not consistently relate to change in vascular function after anti-TNF-α treatment, despite a transient improvement in microvascular endothelial function. Global CVD risk, assessed by using several different algorithms, inversely correlated with microvascular endothelium-dependent function and macrovascular endothelium-independent function in the cross-sectional study, and some classical CVD risk factors improved after anti-TNF-α treatment.

RA is characterized by increased systemic inflammation, which has been hypothesized to affect the vasculature and contribute to accelerated atherosclerosis [30]. However, the present findings suggest that systemic markers of inflammation (ESR, CRP, and DAS28) do not relate to endothelial function in two separate vascular beds, in a cross-sectional or longitudinal study. It is worth noting that a trend was seen for an association between microvascular endothelium-dependent function and CRP in the cross-sectional study; however, given the number of associations that were examined, this could be a chance finding. In the longitudinal study, ΔCRP was associated with change in macrovascular endothelium-dependent function at 2 weeks, but because macrovascular endothelial function did not significantly improve with anti-TNF-α treatment, it is difficult to interpret the implication of this association. In the microvasculature, conflicting associations between endothelial function and disease-related markers of inflammation are reported: one study found an association with TNF-α but not with ESR or CRP [31]; in another, endothelial function was associated with CRP only [10]; and yet another study reported no associations at all [11]. Similarly, a number of studies in the macrovasculature reported no associations between disease-related markers of inflammation and macrovascular endothelium-dependent function [32,33]. Studies that have reported associations present an inconsistent picture, with some finding an association with CRP but not ESR [34], or with DAS28 only [35]. Likewise, the majority of longitudinal studies found no correlations between change in disease-related markers of inflammation and change in microvascular and macrovascular endothelial function in response to antiinflammatory treatment, with only one report of an association for macrovascular endothelium-dependent function [9]. Collectively, these findings suggest that the relation between endothelial function and disease-related markers of inflammation may not be as strong as previously suggested.

The transient improvement in microvascular endothelium-dependent function after treatment with anti-TNF-α resonates with the findings of a previous study. Komai and colleagues [36] found improvements in microvascular endothelium-dependent function after 2 weeks of treatment with anti-TNF-α when assessing microvascular endothelium-dependent function. In addition, although lower than that at 2 weeks, endothelial function was still increased after 6 weeks of treatment [36]. Therefore, it is possible that after an initial improvement, a gradual decrease in microvascular endothelial function occurs between 6 weeks and 12 weeks. Without a 6-week assessment in the current study, this must remain speculation. In contrast, Hansel and colleagues [37] observed no change in microvascular endothelium-dependent function at 2 weeks in RA patients; however, these patients exhibited consistently lower disease-related inflammation than did the present cohort. Given that elevated baseline inflammation was associated with greater change in endothelial function, it is possible that change in endothelial function after treatment is unlikely in patients with low disease-related inflammation, and this may explain the seemingly contrasting findings. In line with our findings, in patients with high baseline disease-related inflammation, short-term improvement in microvascular endothelial function after anti-inflammatory treatment has been reported [38]. Although no long-term follow-up period was applied, the findings support the notion that, in patients with elevated inflammatory markers, acute improvement in microvascular endothelial function is more likely than longer-term improvements after treatment with anti-TNF-α.

Macrovascular endothelium-dependent function did not change in response to 3 months of anti-TNF-α treatment, which is in contrast to previous studies [39-41]. Comparison of the patients included in these studies revealed that average baseline macrovascular endothelium-dependent function was better in the current study than in previous RA patient samples (9.4% versus 2.8% to 7.0%, respectively). Studies that included a healthy control group showed lower baseline macrovascular endothelium-dependent function in the RA patients [39,42]. However, in the present study, the baseline macrovascular endothelium-dependent function of the RA patients was similar to that of healthy control participants (data not shown), therefore making an improvement in response to anti-TNF-α unlikely. Thus, it is possible that treatment with anti-TNF-α may have less impact in RA patients who have similar endothelial function than in healthy individuals.

The majority of individual classical CVD risk factors did not associate with endothelium-dependent function in RA in the cross-sectional study, with age being an exception. In the general population, the association between classical CVD risk factors and endothelial function has been well characterized [43]. To our knowledge, no study of RA patients has examined the impact of classical CVD risk factors on microvascular endothelial function, and only a few studies have examined the effects of classical CVD risk factors on macrovascular endothelial function [32,34,44]. Associations between endothelium-dependent function and lipid levels were present in some [34,44], but not all [32] studies. In the longitudinal study, classical CVD risk factors (SBP, DBP, and HDL cholesterol) improved after reducing RA disease-related inflammation, which is in agreement with other studies [45]. This suggests that inflammation might contribute to the development of classical CVD risk factors in patients with RA. Despite the improvement in classical CVD risk factors with anti-TNF-α therapy, it should be noted that no direct associations were found between the improvement in disease activity and risk factors.

The observation that microvascular but not macrovascular endothelium-dependent function most commonly associated with global CVD risk algorithms and that only microvascular endothelium-dependent function changed after treatment with anti-TNF-α highlights the importance of examining endothelial function in more than one vascular bed. Microvessels make up a much larger proportion of the vasculature than do macrovessels, and may therefore have greater exposure to injurious stimuli [46]. Consequently, it is possible that even small changes in global CVD risk could have a greater effect on microvascular endothelium-dependent function. Therefore, assessments that examine both vascular beds may provide more meaningful clinical information on vascular risk in RA. Even though ED has been associated with clinical end points in patients with cardiovascular disease [4], only one such study with a small sample has reported that high levels of carotid artery intima-media thickness (a subclinical measure of atherosclerosis) are predictive of hard cardiac end points in RA [47]. Therefore, further research is necessary to explore which vascular assessment is the best predictor of cardiac end points in RA patients.

In the macrocirculation, endothelium-independent function was associated with the FRS, Reynolds Risk Score, QRISK 2, insulin resistance, SBP, presence of high cholesterol levels, and hypertension. Evidence indicates that, in healthy individuals with CVD risk factors, abnormalities in endothelium-independent function may occur in the absence of abnormalities in endothelium-dependent function [48]. Further, macrovascular endothelium-independent function, but not macrovascular endothelium-dependent function, was related to a reduction in SBP after 12 and 24 weeks of treatment in patients with hypertension [49]. These findings indicate that CVD risk factors may differentially affect endothelial and smooth muscle cell function, particularly in the macrovasculature. In the current study, endothelium-independent function was significantly lower in patients with hypertension, and some evidence suggests that CVD risk factors like hypertension degrade cyclic guanosine monophosphate (cGMP) [50], a second messenger responsible for the relaxation of vascular smooth muscle cells. In addition, *in vitro *studies have shown that soluble guanylyl cyclase, an enzyme responsible for activating cGMP, has reduced sensitivity to NO in hypertensive rats [51]. This means that, even if adequate NO is released from the endothelial cells, abnormalities in smooth muscle cell signalling could still lead to a reduced vasodilatory response. Thus, examination of vascular function should include combined assessments of endothelial function and smooth muscle cell function.

The strength of the present work was the inclusion of a large cohort of RA patients at baseline, and the longitudinal assessment of patients newly starting anti-TNF-α treatment. Such a study design allowed the investigation of specific predictors of endothelial function at baseline and over a protracted timescale. Another strength of the study was the extensive characterization of CVD risk by using multiple global CVD risk scores (FRS, TCSCORE, TC:HDL SCORE, Reynolds risk score, and QRISK2), along with comprehensive assessment of CVD risk factors, including HOMA and QUICKI. Unfortunately, the longitudinal study did not have a no-treatment RA control group, for obvious ethical reasons. It is acknowledged that the inclusion of a patient group taking stable medication could have strengthened the design of the study, as it would have allowed exploration of potential fluctuations in endothelial function. However, such a control group is likely to have lower baseline levels of disease-related inflammation, making comparisons to patients with active inflammation difficult. Additional limitations include the inability to examine the effects of other disease-modifying antirheumatic drugs, as well as treatment with corticosteroids. Further research is necessary to establish whether alternative anti-inflammatory medications may exert different effects on endothelial function. Furthermore, the use of other biomarkers of inflammation (for example, CD40, interleukin 6) may help to unravel important pathways that may contribute to vascular pathology in RA. Finally, the etiology of RA has a considerable genetic component in particular; certain human leukocyte antigen alleles appear to associate with worse macrovascular endothelium-dependent function in this group of patients [52]. More recent studies have reported that deletion of certain protective genes (such as *CCR5*Δ*32*) can result in lower macrovascular endothelium-dependent function in RA [53]. Thus, further work examining the genetic influence on endothelial function is required.

## Conclusion

In summary, the present findings suggest that global CVD risk algorithms may be better predictors of vascular health than individual CVD risk factors and systemic markers of disease-related inflammation in patients with RA. Furthermore, classical CVD risk and anti-TNF-α treatment appear to differentially impact microvascular and macrovascular endothelial function; therefore, assessments of endothelial function should be performed in both vascular beds. Further studies are needed to confirm whether assessments of vascular function are predictive of long-term CV outcomes in RA.

## Abbreviations

Ach: acetylcholine; anti-TNF-α: anti-tumor necrosis factor-alpha; BMI: body mass index; CRP: C-reactive protein; CVD: cardiovascular disease; CV: coefficient of variation; cGMP: cyclic guanosine monophosphate; DAS28: disease activity score in 28 joints; DBP: diastolic blood pressure; ED: endothelial dysfunction; ESR: erythrocyte sedimentation rate; FMD: flow-mediated dilatation; FRS: Framingham Risk Score; GTN: glyceryl trinitrate-mediated dilatation; HAQ: Health Assessment Questionnaire; HDL: high-density lipoprotein cholesterol; HOMA: homeostasis model assessment; IPAQ: International Physical Activity Questionnaire; LDI: laser Doppler imaging; LDL: low-density lipoprotein cholesterol; NO: nitric oxide; NSAID: nonsteroidal antiinflammatory drug; QUICKI: Quantitative Insulin Sensitivity Check Index; RA: rheumatoid arthritis; SCORE: Systematic Coronary Risk Evaluation; SBP: systolic blood pressure; TC: total cholesterol; TG: triglyceride.

## Competing interests

The authors declare that they have no competing interests.

## Authors' contributions

AS participated in the design of the study, recruited patients, performed the vascular assessments, conducted data analysis, and drafted the manuscript. GK participated in the design of the study, helped with data analysis and in drafting the manuscript. DC participated in the design of the study and helped with data analysis. JVvZ participated in the design of the study, and helped with data analysis and in drafting the manuscript. All authors read and approved the final manuscript.
